# Characterizing the urban diet: development of an urbanized diet index

**DOI:** 10.1186/s12937-022-00807-8

**Published:** 2022-09-09

**Authors:** Ali Cyr-Scully, Annie Green Howard, Erin Sanzone, Katie A. Meyer, Shufa Du, Bing Zhang, Huijun Wang, Penny Gordon-Larsen

**Affiliations:** 1 Department of Nutrition, Gillings School of Global Public Health and School of Medicine, University of North Carolina at Chapel Hill, Chapel Hill, NC USA; 2 Department of Biostatistics, Gillings School of Global Public Health, University of North Carolina at Chapel Hill, 123 W. Franklin Street, CB #8120, Chapel Hill, NC 27514 USA; 3 Carolina Population Center, University of North Carolina at Chapel Hill, 123 W. Franklin Street, CB #8120, Chapel Hill, NC 27514 USA; 4 Nutrition Research Institute, University of North Carolina at Chapel Hill, Kannapolis, NC USA; 5 National Institute for Nutrition and Health, China Center for Disease Control and Prevention, Beijing, China

**Keywords:** China, Diet, Urbanization, Cardiometabolic disease

## Abstract

**Background:**

In recent decades China has experienced rapid urbanization leading to a major nutrition transition, with increased refined carbohydrates, added sweeteners, edible oils, and animal-source foods, and reduced legumes, vegetables, and fruits. These changes have accompanied increased prevalence of cardiometabolic disease (CMD). There is no single dietary measure that summarizes the distinct food changes across regions and levels of urbanization.

**Methods:**

Using a sample of adults (≥18 years) in the 2015 wave of the China Health and Nutrition Survey (CHNS; *n* = 14,024), we selected literature-based candidate dietary variables and tested their univariate associations with overall and within-region urbanization. Using iterative exclusion of select diet-related variables, we created six potential urbanized diet indices, which we examined relative to overall urbanization to select a final urbanized diet index based on a priori considerations, strength of association with urbanization, and minimal missingness. We tested stability of the final urbanized diet index across sociodemographic factors. To examine whether our new measure reflected health risk, we used mixed effects logistic regression models to examine associations between the final urbanized diet index and CMD risk factors – hypertension (HTN), overweight, and type 2 diabetes mellitus (T2DM), adjusting for sociodemographics, overall urbanization, physical activity, and including random intercepts to account for correlation at community and household level.

**Results:**

We identified a final urbanized diet index that captured dietary information unique to consumption of an urbanized diet and performed well across regions. We found a positive association (R^2^ = 0.17, 0.01 SE) between the final urbanized diet index and overall urbanization in the fully adjusted model. The new measure was negatively associated with HTN [OR (95% CI) = 0.93 (0.88–0.99)] and positively associated with T2D [OR = 1.13; 1.05–1.21] in minimally adjusted models, but not in the fully adjusted models.

**Conclusion:**

We derived an urbanized diet index that captured dietary urbanization that was distinct from overall urbanization and performed well across all regions of China. This urbanized diet index provides an alternative to measures of traditional versus urbanized diet that vary across regions due to different cultural dietary traditions. In addition, the new measure is best used in combination with diet quality measures, sociodemographic, and lifestyle measures to examine distinct pathways from urbanization to health in urbanizing countries.

**Supplementary Information:**

The online version contains supplementary material available at 10.1186/s12937-022-00807-8.

## Background

Across the globe, the nutrition transition has shifted food consumption away from traditional diets high in vegetables, carbohydrates, fiber and whole foods, and low in fats to a diet comprising processed foods, saturated fats, added sugar, and little fiber [1, 2]. Through the process of urbanization, adoption of an urban diet is associated with a range of nutrition-related chronic diseases, such as obesity, diabetes, and cardiovascular diseases [2, 3]. However, characterizing an urban diet within and across diverse countries is challenging due to cultural differences in diet across diverse regions. For example, there are clear differences in urbanized diets in rice-based versus wheat-based culinary traditions across China [4].

Thus, there is a great need to develop urbanized diet measures that generalize across regions and cultural traditions. In addition, lower quality nutrition outcomes are more common in urban environments, particularly among low income residents who are sensitive to economic changes that impact access to healthy foods, yet, urban diets also tend to be more diverse, which is generally better, due to access to a wider variety of foods than in rural areas [5]. However, this advantage is not equally distributed across socioeconomic strata, and this urban diet paradox presents another need to better understand the urbanized diet [5]. Adding to this complexity, urbanization brings positive and negative dietary changes, including greater access to wide variety of foods on the one hand and to high fat, energy-dense foods on the other, in addition to a host of other lifestyle changes with potential health impact. Thus, there is value in the development of an urbanized diet measure that can be used in combination with diet quality measures as well as sociodemographic and broader non-dietary urbanization related changes, to allow scientific inquiry regarding the distinct pathways that link urbanization to cardiometabolic and other health outcomes.

While measures of overall urbanization in China capture the multidimensionality of urbanization and move beyond the discrete urban-rural dichotomy [6], such measures do not specifically focus on dietary changes. Given the rapid and differential urbanization changes in dimensions of society with urbanization, there is considerable variation in diet across the full spectrum of urbanization [7]. Yet, there is no single measure of urbanized diet, distinct from a more broad urbanization measures, to quantify changes in diet and diet-related behaviors that have occurred in recent years in China, a country with substantial heterogeneity in diet by geographic region [8, 9]. Given the variation in diet across regions and dietary cultural traditions, the lack of a single urbanized diet measure leaves fundamental gaps in understanding how the dietary urbanization associates with diet-related chronic diseases over time.

China provides a unique opportunity to study changes in diet and its association with cardiometabolic disease (CMD) because of the rapid and differential pace of urbanization that has occurred in recent decades across its regions. With the relatively recent political and social reforms, China has transitioned from a country typically burdened with undernutrition to one with a rapid increase in obesity [10]. Other non-communicable diseases, such as type 2 diabetes mellitus (T2DM), have become more prevalent and are now leading causes of morbidity, disability, and mortality in China [10]. As a whole, diet-related non-communicable diseases have become common, which is of great concern [11]. The China Health and Nutrition Survey (CHNS) is an extensive ongoing, prospective cohort study that began in 1989 with the intent to capture the health impacts of urbanization. The availability of large scale, high-quality CHNS dietary data provides an outstanding exemplar population in which to develop a measure of diet urbanization and investigate associations between diet and CMD.

We aimed to develop an urbanized diet index reflecting the total diet that would generalize across different geographic regions and across varied culinary traditions and consumption patterns. To this end, we sought to generate a single measure of urbanized diet, distinct from but associated with more broad urbanization measures, using a wide variety of diet consumption and diet-related infrastructure variables. We capitalized upon the rapid and differential urbanization across China and the extensive diet data of the CHNS to create an urbanized diet index. We then tested the association between this urbanized diet index with three key CMD risk factors – hypertension (HTN), overweight, and T2DM.

## Methods

### Study participants

The China Health and Nutrition Survey (CHNS) is an ongoing household-based, cohort study. It began in 1989, with surveys completed every 2–4 years until 2015. Prior to 2011, the sample included data from nine provinces (Heilongjiang, Liaoning, Jiangsu, Shandong, Henan, Hubei, Hunan, Guangxi, Guizhou). In 2011 three megacities were added (Beijing, Shanghai and Chonqing) and in 2015 three additional provinces were added (Shaanxi, Yunnan, and Zhejiang). A stratified, multistage, clustered sampling design was used to select households and communities within each province or mega-city. The CHNS captures a variety of geographical areas, levels of economic development, and health indicators [12]. Data were collected through a 7-day survey including collection of biomarker data, 3-day 24-hour diet recall data, eating behaviors, financial resources, and other relevant measures, making the CHNS a robust resource for studying diet urbanization and cardiometabolic disease. The study met the standards for the ethical treatment of participants and was approved by the Institutional Review Boards of the University of North Carolina at Chapel Hill and the National Institute for Nutrition and Health, Chinese Center for Disease Control and Prevention. Participants gave informed consent for participation [12].

**Fig. 1:**
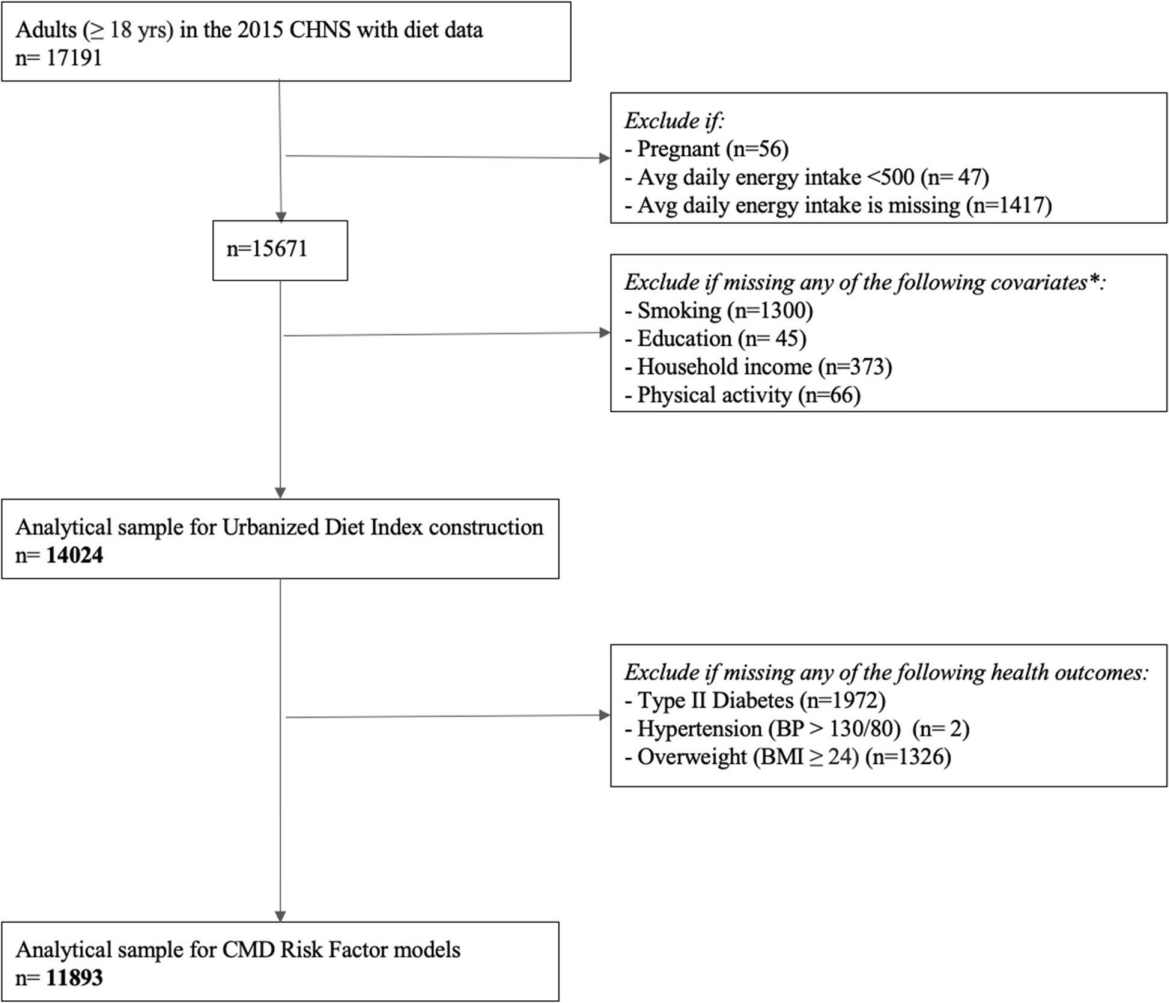
Inclusion and exclusion criteria for the analytic sample, CHNS, 2015. * Individuals may be missing more than one covariate

To develop the urbanized diet index, we used cross-sectional data from all 12 provinces and three megacities in the 2015 wave of the CHNS for all adults, 18 years or older, who had diet data collected in 2015 (*n* = 17,191). All inclusion and exclusion criteria are shown in Fig. 1. We excluded participants who were pregnant, had implausible (< 500 kcals) or missing average daily energy intake (kcal), or had missing covariate or health outcome data.

### Diet data

Dietary intake was measured with 24-hour dietary recalls on three consecutive days. Household food consumption was determined by examining changes in inventory from the beginning to the end of each day, in combination with a weighing and measuring technique. Three-day inventory and dietary recall interviews were randomized to occur between Monday-Sunday and were conducted by trained investigators. Interviewers recorded the type of food, amount consumed, timing of consumption and location of foods consumed, which included food consumed outside the home, for each individual participant. Side dishes, condiments and spices were recorded and weighed at the beginning and end of the food inventory and allocated to household members based on individual 24-hour recall data. Food consumption data was converted to nutritional content using the Chinese Food Composition Table. Other variables were derived from the 24-hour recall data, including percent of daily calories consumed from a variety of foods or food groups (fruit, nuts and seeds, all snack foods, sweet snack foods, eggs, dairy, fried foods, fast food, instant noodles, high fat meat, carbohydrates, fat, animal products, processed foods), and daily averages of number of snacks eaten, number of food groups eaten, sodium intake, and fiber intake. We classified “snacks” to include salty soda cracker or mooncake, sweetened cookies, biscuits, cakes, pastries, and mooncake, nuts (peanut/sunflower seeds/pumpkin seeds, watermelon seeds, and other seeds), chocolate, and potato chips, French fries, and other fried snacks. Sweet snack foods included sweetened cookies, biscuits, cakes, pastries, and mooncake. Processed foods included packaged, frozen, boxed, or bagged foods, as well as oils and condiments that were added during cooking [13].

In addition, we used diet-related data from other parts of the CHNS questionnaire. For example, we used self-reported data on the amount of each type of alcohol consumed each week to generate an indicator for whether an individual consumed any wine. We used data from the household questionnaire to generate diet-related infrastructure indicators of urbanization, including household ownership of refrigerator and/or microwave.

### Health outcome variables

#### Hypertension (HTN)

Blood pressure was measured three times on the right arm after 10 minutes of seated rest by physicians using standard mercury sphygmomanometers [14]. A participant with systolic blood pressure ≥ 130 or diastolic blood pressure ≥ 80 was classified as having HTN based on the ACC/AHA blood pressure guidelines [15]. Additionally, participants who reported a HTN diagnosis or who reported they were currently taking HTN medication were also defined as having hypertension.

#### Overweight

Height was measured without shoes to the nearest 0.1 cm using a portable stadiometer and weight was measured without shoes and in light clothing to the nearest 0.1 kg on a calibrated floor scale. Both were measured by trained anthropometrists and were used to calculate body mass index (BMI). Overweight was defined as having a BMI of 24 kg/m^2^ or greater, based on the Chinese overweight BMI cut point [16].

#### Type II diabetes mellitus (T2DM)

Fasting blood glucose was measured through blood sample collection, after an overnight fast, by health workers, according to standard procedures [17]. T2DM was determined based on individuals having either a fasting blood glucose level of 126 mg/dL or greater [18], the International Diabetes Federations (IDF) criteria for T2DM diagnosis (IDF, 2017 [19]), or a self-reported previous T2DM diagnosis or taking T2DM medications.

#### Urbanization index

To define dietary consumption patterns relative to overall urbanization, we used the urbanization index, a validated multicomponent measure of urbanization in the CHNS that captures rapid and differential urbanization across China [6]. This measure of overall urbanization, calculated at the community level, was derived using a multicomponent continuous scale based on very detailed direct measurement of community contextual measures. The 12 components that comprise this validated index include population density, economic activity, traditional markets, modern markets, transportation infrastructure, sanitation, communications, housing, education, diversity, health infrastructure and social services.

#### Covariates

Age, sex, educational level (highest attained), and smoking history were self-reported. Due to the high correlation between sex and smoking history, we derived a combined variable of sex and smoking status (female never smoker, male never smoker, female ever smoker, male former smoker, male current smoker) for use as a covariate. Region was categorized as North, Central, South, and Megacities. Per capita household income, in Yuan, was derived from individual and household questionnaires from time-use, asset, and economic activity. Average total daily energy intake, in calories, was collected across the repeated 24-hour recall data, which was validated using doubly labelled water. Physical activity was measured using a detailed weekly activity recall which captured occupational, domestic, travel and active leisure activity. Metabolic equivalent of task (MET) hours per week were calculated for each of these categories of physical activity using the Compendium of Physical Activity [20–22]. We calculated a total physical activity index, capturing the sum of occupational, domestic, travel, and active leisure activity MET hours per week. Details on these physical activity variables, including how the METs were calculated can be found elsewhere [23, 24].

### Urbanized diet index development

#### Select dietary variables of interest

First, we selected a broad set of diet-related variables with literature-based evidence for association with urbanization, with the intent to capture the total diet. Second, we examined consistency in the association between these variables with urbanization index, overall and within each region (North, Central, South) and within Megacities, by examining the mean and standard deviation for each continuous dietary measure (e.g., mg of sodium) and percentage for each dichotomous dietary measure (e.g., whether or not an individual consumed wine) by tertile of overall and within-region urbanization index. Third, we determined which variables to move forward, based on (1) association with overall urbanization, (2) consistent association with urbanization across regions, and (3) frequency of consumption > 5%. We used substantive differences in means or percentages across urbanization and region to determine inclusion/exclusion rather than formal statistical testing.

#### Score individual dietary variables

We categorized each individual diet variable for scoring. For uncommonly consumed foods (< 80% of sample are consumers), we created a categorical variable for non-consumers and among the consumers, quartiles of consumption. For commonly consumed foods (≥80% of sample are consumers), we created quintiles of consumption with non-consumers classified in the lowest quintile. Non-continuous variables were scored as dichotomous yes/no consumed or ownership.

To determine scoring of diet variables for the urbanized diet index, we examined the association between each of the candidate diet variables with the overall urbanization index using logistic or multinomial logistic regressions in the following three models: 1) the set of variables from commonly consumed foods (≥80% consumers), 2) the set of variables from infrequently consumed foods (< 80% consumers), and 3) items classified as present/consumed or not present/not consumed. Each of the three sets of diet-related variables are outcome variables and overall urbanization is the exposure variable. For the non-dichotomous dietary variables, we present results as relative risk ratios (RRRs) for associations between each dietary variable and a one standard deviation change in urbanization index, using a referent of the lowest consumer or non-consumer group. For dichotomous variables, we present odds ratios (ORs) for associations between each variable and a one-unit change in overall urbanization index. Using these results, we scored each category of consumption or presence from one to four based on the strength and direction of their association with overall urbanization allowing for the range of the scores to reflect the range of relative risk (e.g., similar scores for similar risk ratios, wider range of scores for wider range of relative risk).

#### Analytical steps to create the urbanized diet index

First, we created six candidate urbanized diet indices for consideration based on inclusion and exclusion of specific food variables. Second, we tested associations for each of the six candidate diet urbanization indices with overall urbanization in unadjusted, age- and sex-adjusted, and fully adjusted (age, sex and smoking, average daily energy intake, region, educational attainment, per capita household income, and physical activity) mixed linear regression models, with random intercepts to account for correlation at the household and community level. We used these model results to select a final diet urbanization measure based on the strength of association with overall urbanization, and degree of missingness.

We then tested whether the final diet urbanization index was stable across varying sociodemographic characteristics using standardized residuals greater or less than two. Using a fully adjusted mixed model linear regression, we classified individuals into two groups, one with individuals who had less accurate prediction of final urbanized diet index (standardized residuals >|2|), and one with individuals who had more accurate prediction of final urbanized diet index (standardized residuals ≤ |2|). The residuals measure the difference between the expected value (prediction from the regression model) and the observed value for each individual. For interpretation purposes, we present standardized residuals (residual divided by the standard deviation of the residuals). We generated average demographic data for each of these two groups and conducted ANOVA and chi-squared tests to determine statistical significance of differences across overall urbanization index, age, sex and smoking status, region, average daily energy intake, educational attainment, per capita household income, and physical activity.

Finally, we tested whether the final diet urbanization index was associated with three key CMD risk variables - HTN, overweight, and T2DM. In a minimally adjusted model, we used mixed effects logistic regression models with urbanized diet index as the exposure, adjusted for age and sex. We used a fully adjusted model, with urbanized diet index as the exposure, adjusted for age, sex/smoking, average daily energy intake, region, educational attainment, per capita household income, physical activity, and random intercepts to account for correlation at community and household levels. In a third set of models, we included all the previous covariates with the addition of the overall urbanization index [6], to compare associations with and without controlling for overall urbanization. We present these results as odds ratios for the association between each of the three CMD outcomes and a one standard deviation change in urbanized diet index.

## Results

Our sample had a slightly higher proportion of women than men (Table 1). Most participants had completed at least some post-primary schooling and lived in the Central or Southern regions of China.

**Table 1:** Descriptive statistics of sample (*N* = 14,024) for generation of Chinese urbanized diet indices, CHNS, 2015

**Age (years) – mean (std)**	**51.45 (15.12)**
Female – N (%)	8050 (57.40)
Urbanization Index^a^– mean (std)	72.52 (17.73)
Educational Attainment – N (%)	
Completed no School	1045 (7.45)
Completed some or all of Primary School	3360 (23.96)
Completed some Post-Primary School	7583 (54.07)
Completed College	2036 (14.52)
Income ^b^ – mean (std)	24.19 (36.30)
Region – N (%)	
North	1587 (11.32)
Central	4645 (33.12)
South	4939 (35.22)
Megacities	2853 (20.34)

^a^Overall urbanization index is a validated multicomponent measure of urbanization in the CHNS (Jones-Smith & Popkin, 2010)

^b^Income is gross per capita household income, in thousands of Yuan

### Select dietary variables of interest

Using literature review, we identified 23 dietary variables (Table 2) as candidates for inclusion in the urbanized diet index. We found consumption of fruit, nuts and seeds, eggs, dairy, fried foods, animal-source foods, high-fat meat, snack foods, processed foods, noodles, fast food and alcohol to be positively associated with urbanization [2, 3, 17, 25, 26]. In addition, snacking behavior, eating at restaurants or food stands, owning a refrigerator or microwave, dietary variety (i.e., food groups consumed), and changes in sodium, fiber, carbohydrate, and fat consumption were found to be positively associated with urbanization [2, 3, 27–29]. Thus, each of these variables was included in the urbanized diet index development process.

**Table 2:** Dietary responses across tertiles of overall urbanization index^a^, CHNS, 2015

	*Urbanization Tertiles*			*Exclusion/Inclusion Rationale*
	***Low*** *29.2–61.2*	***Moderate*** *61.3–82.5*	***High*** *82.6–104.4*	–
Urbanization Index – mean (SD)	50.7 (7.2)	72.9 (6.3)	91.2 (5.2)	–
^b^Drink alcohol - % yes	27.3	27.9	27.9	*Excluded* - No association with urbanization
^b^% kcal from fat – mean (SD)	32.4 (12.7)	35.4 (11.4)	35.1 (10.9)	*Excluded* - No association with urbanization
^b^Sodium (mg) - mean (SD)	3896 (2264)	3974 (2221)	3780 (2092)	*Excluded* - No association with urbanization
^b^Fiber (g) - mean (SD)	10.3 (7.2)	10.8 (7.3)	10.8 (6.9)	*Excluded* - No association with urbanization
^b^Eat fast food – % yes	2.1	2.4	3.2	*Excluded* - Low consumption
^b^Eat instant noodles - % yes	2.0	1.9	2.2	*Excluded*-No association with urbanization, low consumption
Drink wine - % yes	8.4	22.4	31.6	Included - Positive association with urbanization
Own fridge - % yes	85.2	94.0	96.9	Included - Positive association with urbanization
Own microwave - % yes	9.6	39.8	63.0	Included - Positive association with urbanization
Eat fruit - % yes	28.7	40.0	52.25	Included - Positive association with urbanization
Eat nuts/seeds - % yes	11.2	19.4	21.9	Included - Positive association with urbanization
Eat all snack foods - % yes	11.2	17.9	21.5	Included - Positive association with urbanization
Eat sweet snacks - % yes	6.4	12.5	15.5	Included - Positive association with urbanization
Eat eggs - % yes	55.4	70.8	77.2	Included - Positive association with urbanization
Eat dairy - % yes	9.42	19.1	35.8	Included - Positive association with urbanization
Eat away from home - % yes	15.7	30.2	32.0	Included - Positive association with urbanization
Eat fried foods - % yes	18.3	28.5	31.3	Included - Positive association with urbanization
Eat high fat meat - % yes	72.7	85.9	87.0	Included - Positive association with urbanization
Eat animal-source foods - % yes	77.7	90.3	92.6	Included - Positive association with urbanization
Eat processed foods - % yes	93.7	96.3	98.1	Included - Positive association with urbanization
# of snacks - mean (SD)	0.3 (0.5)	0.4 (0.6)	0.6 (0.8)	Included – Positive association with urbanization
# of food groups - mean (SD)	9.9 (2.9)	11.5 (3.2)	12.6 (3.4)	Included - Positive association with urbanization
% kcal from carbs – mean (SD)	55.6 (12.7)	51.1 (11.6)	50.7 (11.0)	Included – Negative association with urbanization

^a^Overall urbanization index is a validated multicomponent measure of urbanization in the CHNS (Jones-Smith & Popkin, 2010)

^b^Variables not included in the urbanized diet index development process

23 variables considered for inclusion in urbanized diet index – drinking alcohol, % of calories from fat, sodium intake and fiber intake excluded for lack of variation with overall urbanization; eating fast food excluded for low consumption (< 5%); eating instant noodles excluded for lack of variation with urbanization and low consumption, carrying 17 variables forward for index development

We conducted preliminary analyses for each to determine patterns of association with overall urbanization index and region. Based on this analysis, we excluded alcohol consumption, percent of calories from fat, sodium intake, and fiber intake, variables which did not vary with overall urbanization. We excluded fast food consumption as it was infrequently consumed (< 5%). We excluded instant noodle consumption, which did not vary with urbanization and was infrequently consumed. Ultimately 17 variables were carried forward that were consistently associated with overall urbanization and region (Supplemental Table 1).

### Score individual dietary variables

**Fig. 2:**
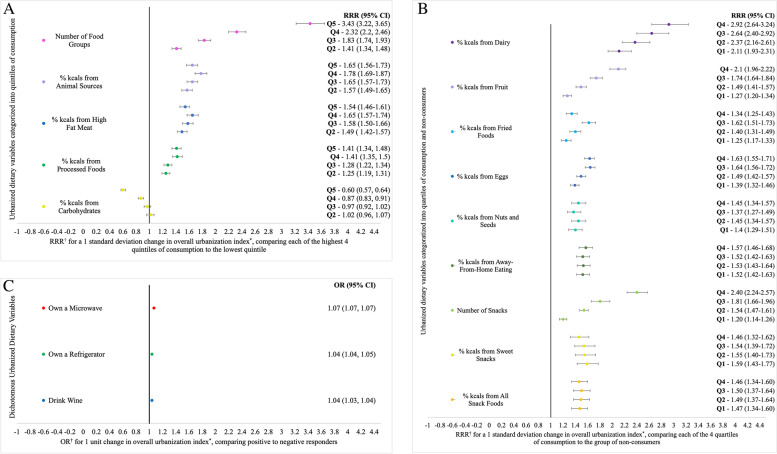
Associations between commonly consumed, uncommonly consumed, dichotomous dietary variables with overall urbanization index^*^, CHNS 2015. **A** Commonly consumed foods (≥ 80% consumption). Relative risk ratios for associations between quintiles of intake and 1-standard deviation change in overall urbanization index^*^ among commonly consumed foods (≥ 80% consumption). ^*^ Urbanization index is a validated multicomponent measure of urbanization in the CHNS [6]. ^†^ The referent group was the lowest quintile, which included non-consumers, in unadjusted multinomial logistic regressions with overall urbanization index as the outcome. **B** Uncommonly consumed foods (< 80% consumers). Relative risk ratios for associations between quartiles of intake and 1-standard deviation change in overall urbanization index ^*^ among uncommonly consumed foods (< 80% consumers). ^*^Urbanization index is a validated multicomponent measure of urbanization in the CHNS [6]. ^†^ Unadjusted multinomial logistic regressions with overall urbanization index as the outcome. **C** Dichotomous dietary variables. Odds ratios for associations between dichotomous dietary variables and 1-unit change in overall urbanization index^*^. ^*^Urbanization index is a validated multicomponent measure of urbanization in the CHNS [6]. ^†^Unadjusted logistic regression models with overall urbanization index as the outcome

Using logistic or multinomial logistic regressions, we examined the association between each of the 17 candidate variables in models based on frequency of consumption and/or ownership. (Fig. 2). Among the frequently consumed foods (Fig. 2A), most were positively associated with overall urbanization, with strongest association for number of food groups, and a negative association between percent of calories from carbohydrates with overall urbanization. Among the less frequently consumed foods (Fig. 2B), we found comparatively stronger positive associations for percent of calories from dairy, fruit and number of snacks with urbanization. All three dichotomous dietary variables (Fig. 2C) were positively associated with urbanization.

We assigned scores to each of the diet-related variables (Table 3), based on the magnitude and pattern of association with overall urbanization (Fig. 2). Scoring was based on consistency and direction of associations (Table 3).

**Table 3:** Scoring of variables based on magnitude and pattern of association with overall urbanization^ab^, CHNS, 2015

*Commonly consumed foods (≥ 80% consumption)*					
	Quintile 1^c^	Quintile 2	Quintile 3	Quintile 4	Quintile 5
Number of Food Groups	0	1	2	3	4
% kcals from Animal Sources	0	1	2	3	2
% kcals from High Fat Meat	0	1	2	3	2
% kcals from Processed Foods	0	1	1	2	2
% kcals from Carbohydrates	4	3	2	1	0
*Uncommonly consumed foods (< 80% consumption)*					
	Non-consumers	Quartile 1	Quartile 2	Quartile 3	Quartile 4
% kcals from Dairy Products	0	1	2	3	4
% kcals from Fruit	0	1	2	3	4
% kcals from Fried Food	0	1	2	3	2
% kcals from Eggs	0	1	1	2	2
Number of Snacks	0	1	2	3	4
*Dichotomous dietary variables*					
	Non-consumers or non-owners	Consumers or owners	–	–	–
% kcals from Nuts & Seeds	0	2	–	–	–
% kcals from Away-from-Home Eating	0	2	–	–	–
% kcals from Sweet Snacks	0	2	–	–	–
% kcals from All Snack Foods	0	2	–	–	–
Own a microwave	0	1	–	–	–
Own a refrigerator	0	1	–	–	–
Drink wine	0	1	–	–	–

^a^Using logistic or multinomial logistic regressions shown in Fig. 2

^b^Overall urbanization index is a validated multicomponent measure of urbanization in the CHNS (Jones-Smith & Popkin, 2010)

^c^Including non-consumers

### Create candidate urbanized diet indices

Each of the 17 candidate variables were carried forward to derive a set of candidate urbanized diet indices (Table 4). We excluded sets of variables for six candidate diet urbanization indices and tested each of these indices to derive a final urbanized diet index. Candidate indices were considered for exclusion if they included variables with considerable missingness or if they had a weaker magnitude of association with urbanization (e.g., RRRs and ORs close to one). Candidate indices were also excluded if they captured duplicate diet features (e.g., numbers of snacks and % of calories from snack foods).

**Table 4:** Urbanized diet index component variables, CHNS, 2015

	*Urbanized Diet Index versions*					
*Dietary Variables*	1	2	3	4	5	6
Drinks Wine – yes/no	X					
Owns a Refrigerator – yes/no	X	X				
Owns a Microwave – yes/no	X	X				
% of calories consumed from All Snack Foods	X	X	X		X	
% of calories consumed from Sweet Snacks	X	X	X	X		
Number of Snacks consumed	X	X	X	X	X	X
% of calories consumed from Fruit	X	X	X	X	X	X
% of calories consumed from Nuts & Seeds	X	X	X	X	X	X
% of calories consumed from Eggs	X	X	X	X	X	X
% of calories consumed from Dairy Products	X	X	X	X	X	X
% of calories consumed from Fried Foods	X	X	X	X	X	X
% of calories consumed from Processed Foods	X	X	X	X	X	X
% of calories consumed from High Fat Meat	X	X	X	X	X	X
% of calories consumed from Animal Source Food	X	X	X	X	X	X
% of calories consumed from Away-from-Home Eating	X	X	X	X	X	X
% of calories consumed from Carbohydrates	X	X	X	X	X	X
Number of Food Groups consumed	X	X	X	X	X	X

Percentages and number of foods consumed were determined based on 3-day averages

Diet Index 1 included all variables, Diet Index 2 excluded wine consumption based on its high missingness, and Diet Index 3, in addition to excluding wine consumption as was done in Diet Index 2, dropped refrigerator and microwave ownership due to low magnitude of association with overall urbanization (Fig. 2). In Diet Indices 4, 5 and 6 we iteratively excluded percent of calories from sweet snack foods, all snack foods, and both snack variables, due to similarity in concept of snacking behavior, from Diet Index 3, which excluded wine consumption, refrigerator, and microwave ownership.

### Select a final urbanized diet index

We compared the associations between each of the six candidate indices (Table 5) with overall urbanization, finding weak positive associations for each. Although the exclusion of the wine consumption variable (Urbanized Diet Index 2) made little difference in the R^2^ relative to Urbanized Diet Index 1, its greater missingness (72%) was less desirable. Each sequential model resulted in minimal difference in R^2^. Based on the strongest correlation with overall urbanization in the fully adjusted model (*R*^2^ = 0.17, 0.01 SE) and low missingness (0.31%), we selected Urbanized Diet Index 2 as the final urbanized diet index.

**Table 5:** Associations between each candidate urbanized diet index and overall urbanization index^a^, CHNS, 2015

						*R*^*2*^ *(SE)*			
*Diet Index*	*Description*	*N*	*Mean (SD)*	*Min*	*Max*	*% Missing*	*Model I*^b^	*Model II* ^c^	*Model III*^d^
*1*	Wine consumption, owning a refrigerator, owning a microwave, daily average % of calories consumed from foods or food groups (fruit, nuts and seeds, all snack foods, sweet snack foods, eggs, dairy products, fried foods, away-from-home eating, high fat meat, carbohydrates, animal-source foods, and processed foods), daily average number of snacks consumed and food groups consumed.	3866	15.76 (6.76)	0	38	72.40	0.18 (0.01)	0.17 (0.01)	0.16 (0.01)
*2*	Diet index 1, excluding wine consumption	13,981	15.36 (6.97)	0	39	0.31	0.18 (0.01)	0.18 (0.01)	0.17 (0.01)
*3*	Diet index 2, excluding owning a refrigerator and microwave	14,024	14.04 (6.72)	0	37	0.0	0.17 (0.01)	0.17 (0.01)	0.16 (0.01)
*4*	Diet index 3, excluding % of calories from all snack foods	14,024	13.72 (6.43)	0	35	0.0	0.16 (0.01)	0.16 (0.01)	0.15 (0.01)
*5*	Diet index 3, excluding % of calories from sweet snack foods	14,024	13.82 (6.49)	0	35	0.0	0.16 (0.01)	0.16 (0.01)	0.15 (0.01)
*6*	Diet index 3, excluding % of calories from all snack foods and sweet snack foods	14,024	13.50 (6.25)	0	33	0.0	0.16 (0.01)	0.16 (0.01)	0.15 (0.01)

Linear mixed model for association of candidate urbanized diet indices and overall urbanization index, accounting for correlations at community and household levels

^a^Urbanization index is a validated multicomponent measure of urbanization in the CHNS (Jones-Smith & Popkin, 2010)

^b^ Model I, unadjusted

^c^ Model II, minimally adjusted for age and sex

^d^ Model III, fully adjusted for age, sex/ smoking status, average daily energy intake (kcals), region, education level, per capita gross household income, physical activity

### Test the stability of the final urbanized diet index across Sociodemographic factors

Using fully adjusted mixed model linear regressions, we tested stability of Urbanized Diet Index 2 across key sociodemographic factors with contrasts for standardized residuals ≤/>|2| using ANOVA and chi-squared tests. We found statistically significant differences in the accuracy of the prediction of urbanized diet index by overall urbanization, age, income, energy intake, region, and educational attainment (Table 6). Individuals with poorer accuracy in prediction of the urbanized diet index had on average a higher overall urbanization index, were younger, had a higher income, and had a lower caloric intake. Similarly, those with poorer accuracy were more likely to be from the Megacities or Central China, as compared to the North and South regions of China, and were more likely to have at least a college education.

**Table 6:** Differences in observed versus predicted^a^ overall urbanization by urbanized diet, CHNS, 2015

	Standardized residuals ≤ |2|	Standardized residuals > |2|
N	13,626	355
^c^Overall Urbanization Index^b^ – mean (SD)	72.4 (17.8)	77.9 (15.1)
^c^Age (years) – mean (SD)	51.6 (15.1)	48.1 (16.4)
^c^Income^d^ – mean (SD)	24.1 (36.3)	28.3 (37.2)
^c^Energy Intake (kcals) – mean (SD)	1844.1 (623.7)	1766.0 (607.6)
^c^Region – N(%)		
North	1559 (11.4)	24 (6.8)
Central	4504 (33.1)	132 (37.2)
South	4824 (35.4)	96 (27.0)
Megacities	2739 (20.1)	103 (29.0)
^c^Educational Attainment – N (%)		
Completed no School	1016 (7.5)	23 (6.5)
Completed some or all of Primary School	3286 (24.1)	64 (18.0)
Completed some Post-Primary School	7385 (54.2)	179 (50.4)
Completed College	1939 (14.2)	89 (25.1)
Sex and Smoking Status – N (%)		
Female Never Smoker	7475 (54.9)	204 (57.5)
Male Never Smoker	1964 (14.4)	42 (11.8)
Female Ever Smoker	339 (2.5)	7 (2.0)
Male Former Smoker	431 (3.2)	15 (4.2)
Male Current Smoker	3417 (25.1)	87 (24.5)
Physical Activity (METs) – mean (SD)	162.4 (172.3)	154.9 (140.0)

^a^Standardized residuals ≤ |2| versus > |2| from adjusted linear mixed model predicting overall urbanization index from urbanized diet index, controlling for age, sex and smoking status, daily energy intake, region, educational attainment, household income, daily physical activity, and accounting for community and household correlations

^**b**^Overall urbanization index is a validated multicomponent measure of urbanization in the CHNS (Jones-Smith & Popkin, 2010)

^c^Statistically significant differences between the two groups

^d^Income is gross per capita household income, in thousands of Yuan

### Test the association between the final urbanized diet index and CMD risk

**Fig. 3:**
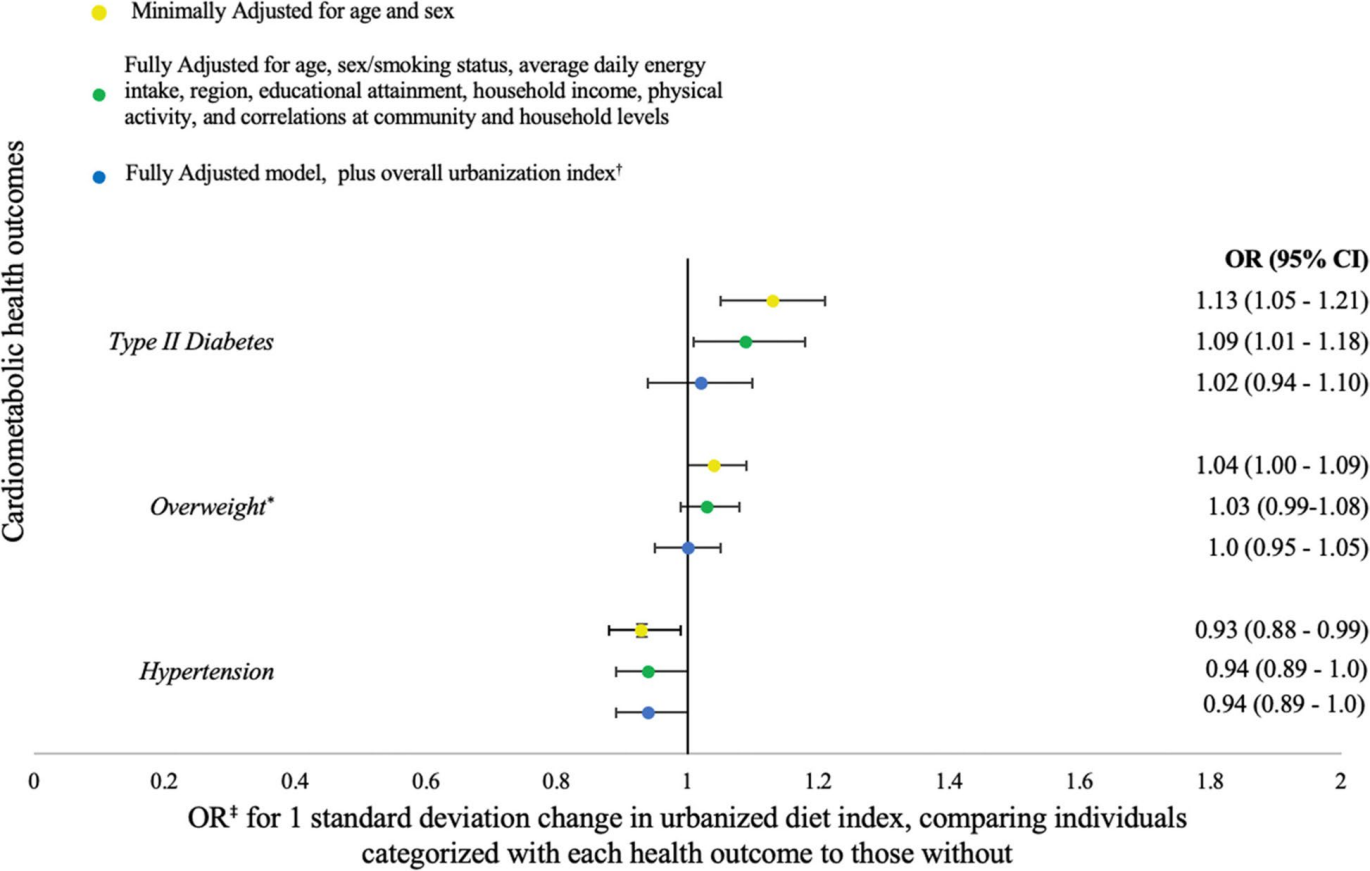
Odds ratios for associations between final Chinese Urbanized Diet Index and HTN, Overweight^*^, T2DM. ^*^Overweight was defined as having a BMI of 24 kg/m^2^ or greater, based on the Chinese overweight BMI cut point (Zhou, 2002). ^†^ The overall urbanization index is a validated multicomponent measure of urbanization in the CHNS [6]. ^‡^Models included logistic regressions with urbanized diet index as the exposure and CMDs as the outcomes

We found a statistically significant association between Urbanized Diet Index 2 and T2DM in the minimally and fully adjusted models, which was attenuated with the addition of overall urbanization. We found a statistically significant association between Urbanized Diet Index 2 and HTN in the minimally adjusted model only. We found no statistically significant association between Urbanized Diet Index 2 and overweight in any of the three models (Fig. 3). We found that sociodemographic factors and lifestyle behaviors were statistically associated with the included CMD risk factors (Supplemental Table 2).

## Discussion

Using 2015 CHNS data we developed a measure that captures dietary urbanization and is positively associated with overall urbanization. Our analysis of associations between each of the individual dietary variables of interest and overall urbanization effectively informed our decisions about inclusion and exclusion of variables, and appropriate scoring of variables for generation of a set of urbanized diet indices. We determined that the urbanized diet index that was the best indicator of diet urbanization included the ownership of a refrigerator and microwave, percent of calories consumed from fruit, nuts and seeds, all snack foods, sweet snacks, eggs, dairy, fried food, away-from-home eating, high fat meat, carbohydrates, animal source foods and processed foods, as well as daily average number of snacks consumed, and daily average number of food groups consumed. The diet index excluded wine consumption. This decision was based on the little impact on the association with overall urbanization upon exclusion of the wine consumption variable, and a large reduction in percent of participants missing data. When adjusting for overall urbanization index, we saw no associations between the urbanized diet index and three CMDs - HTN, overweight and T2DM.

We found a positive association between the urbanized diet index and overall urbanization index (*R*^2^ = 0.17 (0.01 SE)). While this observed association was relatively weak, it is not unexpected given the inclusion of a wide range of diet-related factors in the urbanized diet index. This association suggests that the urbanized diet index does not solely represent urbanization and provides distinct information, specifically about diet. We assessed the stability of the final derived urbanized diet index across sociodemographic factors, finding comparatively lower predictive accuracy by overall urbanization, age, region, energy intake, educational attainment, and income, thus suggesting some variation in ability to discriminate according to sociodemographic factors. The findings for predictive accuracy suggest performance of the new urbanized diet index was much higher or much lower for some individuals than we would anticipate by chance.

Some of the results of our study were unanticipated. We expected that sodium consumption would be an important factor in diet urbanization, but our findings suggest otherwise. In preliminary analysis we saw only slight changes in sodium intake with changing urbanization. This led us to fully exclude the sodium variable from the index development process. This inconsistency may be related to differences in sources of sodium in China based on level of urbanization. While salt was traditionally used as a preservative, this method is being replaced by refrigeration, which becomes more available with increasing urbanization. Yet, high sodium intake persists mainly due to salt added in cooking and increased consumption of high sodium processed foods, especially in higher urbanization areas [30, 31]. In addition, reduced fiber intake is considered a factor in the nutrition transition [1]. However, in preliminary analyses we saw no trend between increasing urbanization and fiber intake, thus the fiber variable was not included in the index development. This may be due to increased consumption of foods high in fiber, like fruit, to make up for reduced consumption of whole grains in high urbanization communities [32]. Further, we considered vegetable and legume consumption as candidate variables for inclusion in the urbanized diet index. However, we found that vegetable and legume consumption did not vary substantially by urbanization level. As such, these foods did not meet our inclusion criteria for index construction.

Additionally, we saw inconsistent associations between each of the snack-related variables (number of snacks consumed, percent of calories from all snack foods, and percent of calories from sweet snack foods) and overall urbanization. While RRRs for associations with overall urbanization steadily increased with increasing number of snacks consumed, RRRs remained stable for percent of calories consumed from all snack foods and sweet snacks, indicating that the behavior of snacking may be a greater factor in diet urbanization than the proportion of calories consumed from snacks. This could be explained by differences in what data was used to construct these dietary variables. For the three-day dietary recall, individuals were asked to classify all foods they ate by meal, specifically breakfast, lunch, dinner or snack. Number of snacks was based on the number of times an individual reported they ate a snack, or a meal outside of breakfast, lunch or dinner. This snack could be comprised of one or more types of food groups. Earlier work in this cohort has suggested, for example, that fruits and beverages are often reported as snacks [33]. However, percent of calories from snacks or sweet snacks were based on food group coding. Therefore, fruits and beverages, which constitute their own food group, would not be included in this category; however, foods like chips and snack cakes are included. This suggests while these measures might be correlated, they are capturing different components of an urbanized diet and therefore could easily result in differences in the association with urbanization.

We expected that HTN, overweight, and T2DM would be positively associated with an urban diet. Yet the positive association for diabetes was no longer statistically significant once we adjusted for overall urbanization. Our urbanized diet index was negatively associated with HTN and positively associated with T2DM in minimally adjusted models, but after adjustment for potential confounders, including sociodemographic factors, the association was no longer statistically significant. The positive association between urbanized diet and HTN, while not statistically significant was similar in the model with control for overall urbanization index. It is important to consider that the index we generated does not measure quality of diet, thus a more urbanized diet does not necessarily indicate a less nutritious diet, which would suggest an association with CMD. For example, our index includes nutritious foods like fruit and nuts and seeds, along with foods like fried foods and high fat meat, which can have opposing impacts on cardiometabolic health. In their examination using 2006 CHNS data, Wang et al. also found inconsistent associations between two diet quality measures and odds of CMD, finding an association between the China Dietary Guideline Index (CDGI) and odds of T2DM in men, but not with the tailored Alternative Healthy Eating Index (tAHEI). Wang et al. found an association between CDGI and abdominal obesity in men, but not with the tAHEI. They found null associations between each diet quality measure and elevated blood pressure and metabolism syndrome in men [34]. As seen in Supplemental Table 2, many sociodemographic factors were statistically associated with CMDs. It is possible that these and other non-dietary factors play a role in shaping CMD risk with urbanization. For example, the urbanization process may influence dietary changes, such as increases in macronutrient intake, energy balance, as well as changing the microbiome, or a chronic stress response resulting from lifestyle and other changes.

Our findings point to a complex dynamic related to urbanization, diet, and the nutrition transition, leaving more work to do to understand how exactly the nutrition transition leads to adverse cardiometabolic health. Better measurement of the urbanized diet may aid in better understanding of this complex dynamic, and our paper is one step in that direction. Traditional diets differ globally and within China, so there is no single set of factors that capture the nutrition transition. Thus, our paper presents a methodology approach that may be broadly applied to different populations, but likely with adaptation and tailoring to local cultural dietary traditions and regional variations.

A main strength of our study is the use of the large CHNS study sample, which includes extensive covariate data allowing us to adjust models for important confounding variables. Three 24-hour diet recalls allowed for the use of average diet data, which increased reliability of our analyses. In addition, the use of household inventories and questionnaires enabled us to use data gathered from multiple sources, further increasing reliability. The diet index includes variables capturing many aspects of an urbanized diet.

There were some limitations to our study and its results. Our findings are limited to the Chinese population and cannot be extrapolated to other populations, as only CHNS data was used. There is also great diversity within China, so we may not have accurately captured an urban diet for all people in China. In addition, the study only utilized 2015 data, thus preventing longitudinal analysis of changes in diet and health outcomes. As is true with all self-reported data, diet recalls may not be fully representative of each participant’s diet, and even 3 days of diet assessment may not be enough to capture some episodically consumed foods. Another minor limitation within the CHNS is that the CHNS survey questions did not specifically delineate between type I and type II DM. However, it can be presumed that most, if not all, cases of DM are type II, as type I DM has very low prevalence in China. As mentioned previously, the measure we developed captured the urbanized diet but does not provide insight into diet quality specifically, so further investigation should be done into the quality of an urbanized diet and associations with disease.

The methods used for this project, and the urbanized diet index itself, add to the resources available to study and better understand dietary urbanization. While there are existing measures that capture overall urbanization, we developed a measure that specifically captures dietary urbanization. The methodology presented can be used to further study changes in diet and its impacts in other urbanizing countries.

## Conclusions

In our cross-sectional study of adults in the 2015 CHNS we developed a measure of diet urbanization that was positively associated with overall urbanization and provides information specifically about diet. Our findings suggest that while urbanized diet may be associated with cardiometabolic risk factors, the magnitude of this association is reduced after adjustment for potential confounders, such as education and income, reflecting non-dietary urbanization-related influences on CMD risk. The derived diet urbanization index can be used to expand upon our understanding of patterns of dietary urbanization and its relation to sociodemographic and health factors in China and potentially across low-middle income countries, and over time. In addition, the new measure is best used in combination with diet quality measures, sociodemographic, and lifestyle measures to examine distinct pathways from urbanization to health in urbanizing countries.

## Supplementary Information

**Additional file 1.**

## Data Availability

Manuscript available as Nutrition Journal pre-print on Research Square prior to publication of final version.  Data available at: http://www.cpc.unc.edu/projects/china/

## Abbreviations

CMD: Cardiometabolic disease
T2DM: Type 2 diabetes mellitus
CHNS: China Health and Nutrition Survey
HTN: Hypertension
BMI: Body mass index
METs: Metabolic equivalent of task
OR: Odds ratio
RRR: Relative risk ratio
