# Supplementary material for: Characterizing the urban diet: development of an urbanized diet index
Source: Nutr J. 2022 Sep 9;21:55. doi: 10.1186/s12937-022-00807-8 (PMC9463720; doi:10.1186/s12937-022-00807-8)
Supplement: Supplementary file 1 — Additional file 1. [file 12937_2022_807_MOESM1_ESM.docx]

| Supp. Table 1. Dietary responses across tertiles of overall urbanization index^*^ by region, CHNS, 2015 | | | | | | | | | | | | |
| --- | --- | --- | --- | --- | --- | --- | --- | --- | --- | --- | --- | --- |
| Region | *North* | | | *Central* | | | *South* | | | *Megacities* | | |
| Urbanization Level^*^ | ***Low*** | ***Mod.*** | ***High*** | ***Low*** | ***Mod.*** | ***High*** | ***Low*** | ***Mod.*** | ***High*** | ***Low*** | ***Mod.*** | ***High*** |
| Overall Urbanization index^†^  – mean (SD) | 53.1 (6.2) | 71.6 (6.2) | 91.0 (5.2) | 49.9 (6.5) | 71.8 (5.4) | 91.3 (5.8) | 50.9 (8.2) | 72.8 (6.6) | 91.6 (5.0) | 49.2 (5.8) | 75.6 (6.2) | 90.7 (4.8) |
| Drink wine - % yes | 2.6 | 3.4 | 19.3 | 6.1 | 21.8 | 30.4 | 13.2 | 20.2 | 31.8 | 7.45 | 34.2 | 38.5 |
| Own fridge - % yes | 91.2 | 94.4 | 97.2 | 84.9 | 95.8 | 97.8 | 82.1 | 90.2 | 94.0 | 89.1 | 99.2 | 99.1 |
| Own microwave - % yes | 10.0 | 19.4 | 58.1 | 9.4 | 43.6 | 65.7 | 10.3 | 25.6 | 47.1 | 7.1 | 71.5 | 79.6 |
| Eat fruit - % yes | 33.9 | 57.7 | 57.7 | 28.7 | 36.4 | 53.5 | 30.3 | 39.8 | 43.8 | 11.9 | 41.0 | 58.1 |
| Eat nuts/seeds - % yes | 11.9 | 41.0 | 58.1 | 15.1 | 20.1 | 26.4 | 10.2 | 18.0 | 16.8 | 3.8 | 22.8 | 24.0 |
| Eat all snack foods - % yes | 9.8 | 17.9 | 15.7 | 16.5 | 20.8 | 23.2 | 6.6 | 12.3 | 14.1 | 7.6 | 24.8 | 29.9 |
| Eat sweet snacks - % yes | 5.6 | 10.8 | 10.9 | 10.0 | 14.6 | 16.5 | 3.4 | 8.5 | 8.6 | 3.2 | 18.3 | 23.5 |
| Eat eggs - % yes | 70.1 | 69.6 | 77.0 | 59.9 | 76.5 | 80.4 | 46.1 | 64.1 | 70.7 | 47.1 | 75.8 | 81.4 |
| Eat dairy - % yes | 5.9 | 20.7 | 28.8 | 12.4 | 20.2 | 36.3 | 8.5 | 11.0 | 17.3 | 5.6 | 34.1 | 56.9 |
| Eat away from home- % yes | 8.2 | 15.2 | 19.6 | 16.9 | 27.0 | 27.4 | 18.6 | 35.4 | 41.9 | 9.9 | 29.2 | 29.8 |
| Eat fried foods - % yes | 20.4 | 17.9 | 34.6 | 18.7 | 33.5 | 34.9 | 18.4 | 23.7 | 20.7 | 11.3 | 33.6 | 38.1 |
| Eat high fat meat - % yes | 65.2 | 64.6 | 71.9 | 58.4 | 78.3 | 83.4 | 89.0 | 93.5 | 94.0 | 84.5 | 89.4 | 87.9 |
| Eat animal-source food-% yes | 71.3 | 73.5 | 82.6 | 64.6 | 83.5 | 88.3 | 92.9 | 96.6 | 98.0 | 86.7 | 93.5 | 93.8 |
| Eat processed foods - % yes | 91.5 | 93.7 | 97.6 | 97.0 | 96.4 | 98.2 | 90.4 | 96.2 | 97.4 | 96.6 | 97.3 | 99.0 |
| # of snacks - mean (SD) | 0.3  (0.5) | 0.7  (0.7) | 0.8  (0.9) | 0.3  (0.6) | 0.4  (0.6) | 0.7  (0.8) | 0.3  (0.5) | 0.4  (0.6) | 0.5  (0.6) | 0.1  (0.2) | 0.5  (0.7) | 0.8  (0.8) |
| # of food groups - mean (SD) | 10.3  (2.7) | 11.0  (2.9) | 12.6  (3.4) | 10.7  (2.9) | 11.8  (3.2) | 13.0  (3.2) | 9.3  (2.8) | 10.8  (3.2) | 11.4  (3.0) | 8.6  (2.0) | 12.4  (3.2) | 13.5  (3.5) |
| % kcal from carbs– mean (SD) | 53.9  (10.3) | 53.9 (10.1) | 52.9  (9.4) | 59.7  (12.4) | 52.5  (12.2) | 52.6  (11.3) | 52.2 (12.7) | 50.1 (11.5) | 49.1 (11.2) | 53.2  (12.7) | 50.2 (10.7) | 50.1 (10.8) |
| ^‡^Drink alcohol - % yes | 27.3 | 24.7 | 28.8 | 25.7 | 29.6 | 29.2 | 29.8 | 28.1 | 31.0 | 24.7 | 25.8 | 23.3 |
| ^‡^% kcal from fat – mean (SD) | 33.9  (10.7) | 33.1 (10.3) | 33.2 (9.6) | 28.4  (12.4) | 33.8  (11.9) | 33.2  (10.8) | 35.6  (12.4) | 36.9  (11.4) | 37.0  (11.3) | 35.7  (13.0) | 35.5  (10.6) | 35.3  (10.8) |
| ^‡^Sodium(mg) - mean (SD) | 4044  (2190) | 4055  (2229) | 4235  (2335) | 4230  (2372) | 4233  (2270) | 4007  (2095) | 3491  (2089) | 3802  (2182) | 3588  (1994) | 3757  (2305) | 3881  (2179) | 3637  (2061) |
| ^‡^Fiber(g) - mean (SD) | 9.8  (5.8) | 10.4  (7.4) | 10.9  (6.3) | 11.8  (7.9) | 10.9  (6.2) | 11.1  (6.6) | 9.5  (6.5) | 11.1  (8.5) | 11.0  (7.9) | 7.8  (7.3) | 10.0  (6.3) | 10.4  (6.0) |
| ^‡^Eat fast food – % yes | 0.8 | 0.3 | 2.1 | 2.7 | 2.2 | 2.5 | 1.8 | 3.0 | 1.7 | 2.4 | 2.5 | 5.7 |
| ^‡^Eat instant noodles - % yes | 3.0 | 3.4 | 1.8 | 3.0 | 3.0 | 2.1 | 0.5 | 0.8 | 0.9 | 1.4 | 1.7 | 3.9 |

^*^ North region, tertiles defined as Low 41.5-60.4, Moderate 62.6-82.1, and High 82.6-102.1; Central region, tertiles defined as Low 31.5-60.3, Moderate 61.3-81.9, and High 82.9-103.9; South region, tertiles defined as Low 29.2-61.2, Moderate 61.4-82.6, and High 83.5-104.4; Megacities, tertiles defined as Low 42.1-58.5, Moderate 61.6-81.8, and High 82.6-100.7.

^†^ Overall urbanization index is a validated multicomponent measure of urbanization in the CHNS (Jones-Smith & Popkin, 2010).

^‡^Variables not included in the urbanized diet index development process.

23 variables considered for inclusion in urbanized diet index – drinking alcohol, % of calories from fat, sodium intake and fiber intake excluded for lack of variation with overall urbanization; eating fast food excluded for low consumption (<5%); eating instant noodles excluded for lack of variation with urbanization and low consumption, carrying 17 variables forward for index development.

| Supp. Table 2 |  | | Odds ratios for associations between covariates and HTN, Overweight^*^, T2DM. | | | | | | |
| --- | --- | --- | --- | --- | --- | --- | --- | --- | --- |
|  | |  | | *Hypertension* | | *Overweight* | | *Diabetes Mellitus* | |
|  | |  | | *OR* | *95% CI* | *OR* | *95% CI* | *OR* | *95% CI* |
| Age (1 year change) | |  | | 1.06 | 1.05 - 1.06 | 1.01 | 1.00 – 1.01 | 1.05 | 1.04 – 1.06 |
| Sex/smoking | | Women – Never Smoked | | Ref | Ref | Ref | Ref | Ref | Ref |
|  | | Men – Never Smoked | | 2.00 | 1.74 – 2.30 | 1.35 | 1.20 – 1.52 | 1.24 | 1.01 – 1.52 |
|  | | Female – Ever Smoked | | 0.76 | 0.57 – 1.02 | 0.80 | 0.63 – 1.03 | 1.45 | 1.00 – 2.10 |
|  | | Men – Former Smoker | | 2.44 | 1.81 – 3.31 | 1.27 | 1.02 – 1.59 | 1.43 | 1.04 – 1.98 |
|  | | Men – Current Smoker | | 1.90 | 1.70 – 2.12 | 0.94 | 0.85 – 1.03 | 1.30 | 1.09 – 1.54 |
|  | |  | |  |  |  |  |  |  |
| Energy Intake (1 std change in kcal) | | | | 1.05 | 1.00 – 1.10 | 1.13 | 1.08 – 1.18 | 1.02 | 0.95 – 1.10 |
| Region | | North | | Ref | Ref | Ref | Ref | Ref | Ref |
|  | | Central | | 0.88 | 0.70 – 1.11 | 0.67 | 0.57 – 0.78 | 0.80 | 0.65 – 0.99 |
|  | | South | | 0.55 | 0.44 – 0.69 | 0.52 | 0.44 – 0.61 | 0.69 | 0.56 – 0.86 |
| Education | | Completed no School | | Ref | Ref | Ref | Ref | Ref | Ref |
|  | | Completed some or all Primary School | | 0.93 | 0.76 - 1.14 | 1.05 | 0.89 – 1.24 | 1.42 | 1.07 – 1.89 |
|  | | Completed some Post-Primary School | | 0.80 | 0.65 - 0.98 | 0.95 | 0.80 – 1.12 | 1.51 | 1.12 – 2.03 |
|  | | Completed College | | 0.62 | 0.48 – 0.80 | 0.66 | 0.53 – 0.82 | 1.09 | 0.74 – 1.60 |
| Household Income (1 std change) | | | | 1.02 | 0.97 - 1.07 | 0.98 | 0.94 – 1.02 | 0.96 | 0.88 – 1.05 |
| Physical Activity (1 std change in MET hrs/wk) | | | | 0.99 | 0.94 – 1.04 | 0.97 | 0.93 – 1.01 | 0.96 | 0.88 – 1.05 |
| Urbanization Index^†^ (1 std change in Urbanizaition Index) | | | | 1.00 | 0.91 – 1.09 | 1.16 | 1.09 – 1.24 | 1.31 | 1.19 – 1.44 |

^*^Overweight was defined as having a BMI of 24 kg/m^2^ or greater, based on the Chinese overweight BMI cut point (Zhou, 2002).

^†^ The overall urbanization index is a validated multicomponent measure of urbanization in the CHNS (5).

Models included logistic regressions with urbanized diet index as the exposure and CMDs as the outcomes, controlling for all covariates listed, and accounting for correlations at the community and household levels.
